# Supplementary material for: Genome-wide analysis of the polyamine oxidase gene family in wheat (Triticum aestivum L.) reveals involvement in temperature stress response
Source: PLoS One. 2020 Aug 31;15(8):e0236226. doi: 10.1371/journal.pone.0236226 (PMC7458318; doi:10.1371/journal.pone.0236226)
Supplement: S1 Text — Polyamine oxidase (PAOs) protein sequences from A. thaliana and O. sativa. Z. mayz, B. distachyon and H. vulgare that used for the identification of wheat, T. urartu and Ae. tauschii PAOs. (DOCX) [file pone.0236226.s001.docx]

**S1 Text**

>sp|Q0J290|PAO7_ORYSJ Polyamine oxidase 7 OS=Oryza sativa subsp. japonica OX=39947 GN=PAO7 PE=1 SV=1

MTKPTTMAIFLSIVLLSMAQLPSLVAGTGRPRVIIIGAGISGISAGKRLSEAGITDILIL

EATDHIGGRMHKQRFAGVNVEIGANWVEGVNGEKMNPIWPIVNSTLKLRNFLSDFDSLAQ

NVYKDGGLCDAAYVQKRIDLADEADKSGENLSATLHPSGRDDMSILSMQRLNNHLPNGPS

SPVDMVVDYFTYDYEFAEPPRVTSLRNTVPLPTFTDFGDDNYFVADQRGYEAVVYYLAGQ

YLEADKSGNIVDARLQLNKVVREISYSSTGVTVKTEDNSTYQADYVMVSASLGVLQSDLI

QFKPQLPSWKILAIYQFDMAVYTKIFVKFPKKFWPEGAGREFFLYASTRRGYYGVWQEFE

KQYPDANVLLVTVTDEESRRIEQQPDSQTKAEIMEVVRSMFPDEDVPDATDILVPRWWSD

RFFQGSFSNWPIGVSRYEHDQLRAPVGRVYFTGEHTSERYNGYVHGAYLAGIYA

>sp|Q9SU79|PAO5_ARATH Probable polyamine oxidase 5 OS=Arabidopsis thaliana OX=3702 GN=PAO5 PE=1 SV=1

MAKKARIVIIGAGMAGLTAANKLYTSSNNTFELSVVEGGSRIGGRINTSEFSSEKIEMGA

TWIHGIGGSPVYRIAKETGSLVSDEPWECMDSTIDKAKTFAEGGFEIEPSIVESISGLFT

ALMELAQGKEISQSDADLSRLAHIYETATRVCSKGSSTSVGSFLKSGFDAYWDSISNGGE

EGVKGYGKWSRKSLEEAIFTMFSNTQRTYTSADELSTLDFAAESEYQMFPGEEITIAKGY

LSVIHHLASVLPQGVIQLNRKVTKIEWQSNEVKLHFSDGSVVFADHVIVTVSLGVLKAGI

ETDAELFSPPLPDFKSDAIRRLGYGVVNKLFVEMSQRKFPSLQLVFDREDSEFRFVKIPW

WMRRTATITPIHSNSKVLLSWFAGKEALELEKLTDEEIKDAVMTTISCLTGKEVKNDTAK

PLTNGSLNDDDEAMKITKVLKSKWGSDPLFRGSYSYVAVGSSGDDLDAMAEPLPKINKKV

GQVNGHDQAKVHELQVMFAGEATHRTHYSTTHGAYYSGLREANRLLKHYKCNF

>sp|Q9LYT1|PAO3_ARATH Polyamine oxidase 3 OS=Arabidopsis thaliana OX=3702 GN=PAO3 PE=1 SV=1

MESGGKTNRQLRKAICVSTDEKMKKKRSPSVIVIGGGMAGISAARTLQDASFQVVVLESR

DRIGGRVHTDYSFGFPVDLGASWLHGVCKENPLAAVIGRLGLPLYRTSGDNSVLYDHDLE

SYALFDKAGNQVSQELVTKVGENFEHILEEICKVRDEQDEDMSIAQAFSIVFKRNPELRL

EGLAHNVLQWYLCRMEGWFAADAETISAKCWDQEELLPGGHGLMVRGYRPVINTLSKGLD

IRLSHRITKISRRYSGVKVTTEKGDTFVADAAVIALPLGVLKSGMITFEPKLPQWKQEAI

NDLGVGIENKIILNFDNVFWPNVEFLGVVAETSYGCSYFLNLHKATSHPVLVYMPAGQLA

RDIEKKSDEAAANFAFSQLQKILPDASSPINYLVSRWGSDINSLGSYSYDIVNKPHDLYE

RLRVPLDNLFFAGEATSSSYPGSVHGAYSTGVLAAEDCRMRVLERYGELEHEMEEEAPAS

VPLLISRM

>sp|Q7X809|PAO3_ORYSJ Polyamine oxidase 3 OS=Oryza sativa subsp. japonica OX=39947 GN=PAO3 PE=1 SV=2

MANNSSYGENVRRKSHTPSAIVIGSGFAGIAAANALRNASFEVVLLESRDRIGGRIHTDY

SFGFPVDLGASWLHGVCEENPLAPIIGRLGLPLYRTSGDDSVLFDHDLESYALYDTKGHQ

VPQELVEKIGKVFETILEETGKLREETKEDISIAKAIAIVMERNPHLRQEGIAHDVLQWY

LCRMEGWFATDADAISLQGWDQEVLLPGGHGLMVRGYRPVINTLAKGLDIRLGHRVVEIV

RHRNRVEVTVSSGKTFVADAAVIAVPLGVLKANTIKFEPRLPEWKEEAIRELSVGVENKI

ILHFSEVFWPNVEFLGVVSSTTYGCSYFLNLHKATGHPVLVYMPAGRLACDIEKLSDEAA

AQFAFSQLKKILPNAAEPIHYLVSHWGSDENTLGSYTFDGVGKPRDLYEKLRIPVDNLFF

AGEATSVQYTGTVHGAFSTGLMAAEECRMRVLERFRELDMLEMCHPAMGEQTATVSVPLL

ISRL

>sp|Q5NAI7|PAO1_ORYSJ Polyamine oxidase 1 OS=Oryza sativa subsp. japonica OX=39947 GN=PAO1 PE=1 SV=1

MVAKKPRVVVVGAGISGLAAAHRLCGAGGDRFEVAVVEAGDRVGGRILTSEFAGHRVEMG

ATWVQGVVGSPVYALARDAGALGEEEGRGLPYERMDGFPDRVLTVAEGGEVVDADTVAGP

IEELYRGMMEAARAGEAGGGGGVEEYLRRGLRAYQAARSAGGGGGGGKELEEVDEALLAM

HINRERTDTSADDLGDLDLTAEGEYRDFPGEHVTIPGGYSRVVERLAAALPPGTVRLGLR

LRRLKWGGTPVRLHFADGAPPLTADHVILTVSLGVLKASLGNKDTAGVGAAAIAFDPPLP

PFKREAVARLGFGVVNKLFMEVEAVAPSEPEDVAGVQPAAAGFPFLHMAFRGHVSKIPWW

MRGTESICPVHAGSTVALAWFAGREAAHLESLPDDDVIRGAHATLDSFLPAAPRWRVRRI

KRSGWATDPLFLGSYSYVAVGSSGDDLDRMAEPLPRGPDAAADERPPSPRLLFAGEATHR

THYSTTHAAYLSGVREANRLLQHYRGGANHTT

>sp|Q0J954|PAO5_ORYSJ Polyamine oxidase 5 OS=Oryza sativa subsp. japonica OX=39947 GN=PAO5 PE=1 SV=1

MDQPSNGFAAGGLFLRHIDGQNASPPSVIVIGGGISGIAAARALSNASFKVTLLESRDRL

GGRVHTDYSFGCPIDMGASWLHGVCNENSLAPLIRLLGLRLYRTSGDNSVLYDHDLESYA

LFDKDGRQVPQEIVTKVGETFEKILKETVKVRAEHEDDMPLIQAISIVLDRNPHLKLDGL

QYEVLQWCICRLEAWFATDVDNISLKNWDQEHVLTGGHGLMVHGYDPVIKALAQDLDIHL

NHRVTKIIQRYNKTIVCVEDGTSFVADAAIITVPLGVLKANIIKFEPELPDWKLSSISDL

GIGIENKIALRFNSVFWPNVEVLGRVAPTSNACGYFLNLHKATGHPVLVCMVAGRFAYEF

EKLSDEESVNFVMSQLKKMLPGATEPVQYLVSRWGTDPNSLGSYSCDLVGKPADLYERFC

APVGNLFFAGEAACIDHSGSVHGAYSSGIVAAEDCRRHLSTQLGISDLFQVGKIIMREEM

TEVMVPFQISRL

>sp|Q7XR46|PAO4_ORYSJ Polyamine oxidase 4 OS=Oryza sativa subsp. japonica OX=39947 GN=PAO4 PE=1 SV=1

MDPNSLKTGGLLLPTIERQCASPPSVIVIGGGISGVAAARALSNASFEVTVLESRDRVGG

RVHTDYSFGCPIDMGASWLHGVCNENSLAPLIGYLGLKLYRTSGDNSVLYDHDLESYALF

DKAGHQVSKETVAKVEETFERILDETVKVRDEQEHDMPLLQAISLVLERHPHLKLQGIDD

QVLQWCVCRLEAWFAADADEISLKNWDQEHVLTGGHGLMVNGYYPIIQALAQGLDIRLNQ

RVTKIARQFNGVTVTTEDGTSYSADACIITVPLGVLKANIIKFEPELPSWKSSAIADLGV

GIENKIAMHFDTVFWPNVEVLGMVGPTPKACGYFLNLHKATGNPVLVYMAAGRFAQEVEK

LSDKEAVDLVMSHLKKMLPDATEPTKYLVSRWGSDPNSLGSYSCDLVGKPADVSARFAAP

VENLYFAGEAASADHSGSVHGAYSSGIAAADECRKRILMQKGIPDLVQVKAYEEMAGVIA

PLQICRT

>sp|Q9SKX5|PAO2_ARATH Polyamine oxidase 2 OS=Arabidopsis thaliana OX=3702 GN=PAO2 PE=1 SV=1

MESRKNSDRQMRRANCFSAGERMKTRSPSVIVIGGGFGGISAARTLQDASFQVMVLESRD

RIGGRVHTDYSFGFPVDLGASWLHGVCKENPLAPVIGRLGLPLYRTSGDNSVLYDHDLES

YALFDMDGNQVPQELVTQIGVTFERILEEINKVRDEQDADISISQAFSIVFSRKPELRLE

GLAHNVLQWYVCRMEGWFAADAETISAKCWDQEELLPGGHGLMVRGYRPVINTLAKGLDI

RVGHRVTKIVRRYNGVKVTTENGQTFVADAAVIAVPLGVLKSGTIKFEPKLPEWKQEAIN

DLGVGIENKIILHFEKVFWPKVEFLGVVAETSYGCSYFLNLHKATGHPVLVYMPAGQLAK

DIEKMSDEAAANFAVLQLQRILPDALPPVQYLVSRWGSDVNSMGSYSYDIVGKPHDLYER

LRVPVDNLFFAGEATSSSFPGSVHGAYSTGLMAAEDCRMRVLERYGELDLFQPVMGEEGP

ASVPLLISRL

>sp|A0A0P0XM10|PAO6_ORYSJ Polyamine oxidase 6 OS=Oryza sativa subsp. japonica OX=39947 GN=PAO6 PE=2 SV=1

MTKPTTMAIFLVLALSIAQLLPSLVAGTGRPRVIIVGAGISGISAGKRIWEAGIADVLIL

EATDRIGGRMHKQSFAGVNVEIGANWVEGVNGEKKNPIWPIVNSTLKLRSFRSDFDSLAQ

NVYKDGGLCDEAYVQKRMDRADEVDKSGENLSATLHPSGRDDMSILSMQRLNDHLPNGPS

SPVDMAVDYFTYDYEFAEPPRVTSLQNTVPLPTFTDFGDDTYFVADQRGYESVVHHLAGQ

YLNADKSGNIADARLKLNKVVREISYSSTGVTVKTEDNSTYQADYVMVSASLGVLQSDLI

QFKPQLPSWKILAIYQFDMAVYTKIFVKFPKKFWPEGAGREFFLYASTRRGYYGVWQEFE

KQYPDANVLLVTVTDEESRRIEQQPDSQTKAEIMEVVRCMFPDEDVPDATDILVPRWWSD

RFFRGSFSNWPIGVSRYEYDQLRAPVGRVYFTGEHTSERYNGYVHGAYLAGIDSAEILIN

CAQKKMCKYNVGGKHG

>sp|Q8H191|PAO4_ARATH Probable polyamine oxidase 4 OS=Arabidopsis thaliana OX=3702 GN=PAO4 PE=1 SV=1

MDKKKNSFPDNLPEGTISELMQKQNNVQPSVIVIGSGISGLAAARNLSEASFKVTVLESR

DRIGGRIHTDYSFGCPVDMGASWLHGVSDENPLAPIIRRLGLTLYRTSGDDSILYDHDLE

SYGLFDMHGNKIPPQLVTKVGDAFKRILEETEKIRDETANDMSVLQGISIVLDRNPELRQ

EGMAYEVLQWYLCRMEAWFAVDANLISLKCWDQDECLSGGHGLMVQGYEPVIRTIAKDLD

IRLNHRVTKVVRTSNNKVIVAVEGGTNFVADAVIITVPIGVLKANLIQFEPELPQWKTSA

ISGLGVGNENKIALRFDRAFWPNVEFLGMVAPTSYACGYFLNLHKATGHPVLVYMAAGNL

AQDLEKLSDEATANFVMLQLKKMFPDAPDPAQYLVTRWGTDPNTLGCYAYDVVGMPEDLY

PRLGEPVDNIFFGGEAVNVEHQGSAHGAFLAGVSASQNCQRYIFERLGAWEKLKLVSLMG

NSDILETATVPLQISRM

>sp|Q9FNA2|PAO1_ARATH Polyamine oxidase 1 OS=Arabidopsis thaliana OX=3702 GN=PAO1 PE=1 SV=1

MSTASVIIIGAGISGISAAKVLVENGVEDVLILEATDRIGGRIHKQNFGDVPVELGAGWI

AGVGGKESNPVWELASRFNLRTCFSDYTNARFNIYDRSGKIFPTGIASDSYKKAVDSAIL

KLKSLEAQCSGQVAEEAPSSPKTPIELAIDFILHDFEMAEVEPISTYVDFGEREFLVADE

RGYECLLYKMAEEFLVTSHGNILDYRLKLNQVVREVQQSRNGVVVKTEDGSVYEANYVIV

SASIGVLQSDLLSFQPLLPRWKTEAIQKCDVMVYTKIFLKFPQCFWPCGPGQEFFIYAHE

QRGYFTFWQHMENAYPGSNILVVTLTNEQSKRVEAQSDQETMKEAMSVLRDMFGATIPYA

TDILVPRWWNNRFQRGSYSNYPMISDNQLLQNIKAPVGRIFFTGEHTSEKFSGYVHGGYL

AGIDTSKSLLEEMKQSLLLQPLLAFTESLTLTHQKPNNSQIYTNVKFISGTS

>ZmPAO1-Zm00001d024281_T001-apoplast

MSSSPSFGLLALAAVLLALNLAQHGSLAATVGPRVIVVGAGMSGISAAKRLSEAGITDLL

ILEATDHIGGRMHKTNFAGINVELGANWVEGVNGGKMNPIWPIVNSTLKLRNFRSDFDYL

AQNVYKEDGGVYDEDYVQKRIELADSVEEMGEKLSATLHASGRDDMSILAMQRLNEHQPN

GPATPVDMVVDYYKFDYEFAEPPRVTSLQNTVPLATFSDFGDDVYFVADQRGYEAVVYYL

AGQYLKTDDKSGKIVDPRLQLNKVVREIKYSPGGVTVKTEDNSVYSADYVMVSASLGVLQ

SDLIQFKPKLPTWKVRAIYQFDMAVYTKIFLKFPRKFWPEGKGREFFLYASSRRGYYGVW

QEFEKQYPDANVLLVTVTDEESRRIEQQSDEQTKAEIMQVLRKMFPGKDVPDATDILVPR

WWSDRFYKGTFSNWPVGVNRYEYDQLRAPVGRVYFTGEHTSEHYNGYVHGAYLSGIDSAE

ILINCAQKKMCKYHVQGKYD

>BdPAO1-BRADI_3g25580v3

MMKPSVAIVLLLVVAEYASLATAAGPKVIIVGAGMSGISAGKRLSDARISDFMILEATDR

IGGRIHKTKFAGVNVEMGANWVEGVNGKEKNPIWTMANSTGGLNLRTFRSDFDHLASNTY

KQDGGLYDDKFVEKIIERMDEVEESGTKLAGTLHLSGQEDMSVMAMQRLNDHMPTGPARP

VDMVIDYYQHDFEFAEPPRVTSLQNTVPLPTFDNFGDDVYFVADQRGFESVVYHVAGQYL

KTDKATGAIVDPRLKLNTVVREITHFPSGVTVKTEDNNVYKADYVMVSASLGVLQSELIR

FRPQLPSWKILSIYQFDMAVYTKIFLKFPRSFWPVGPGREFFLYASGRRGYYPVWQQFEK

QYPGSNVLLVTVTDDESRRIEQQPDNQTMAEAVAVLRKMFPGADVPDATKILVPRWWSNK

FYKGSFSNWPIGVNRYEYDLIRAPVGRVYFTGEHTSENYNGYVHGAYLAGIDSADVLINC

AKKKLCKYHVRGKHE

>BdPAO2-BRADI_5g22220v3-N1-acetylspermine:oxygen_oxidoreductase_(3-acetamidopropanal-forming)_activity-peroxisome

MAKNSSFGENVRRNPPTPSAIVIGGGFAGIAAANALRNASFEVVLLESRDRIGGRVHTDY

SFGFPVDLGASWLHGVCEENPLAPIIGRLGLPLYRTSGDDSVLFDHDLESYALYDTNGHQ

VPQEFVEKMGKVFEAILEETGKLREETEEDISIAKAIAIVMERNPHLRQEGMAHDVLQWY

LCRMEGWFATDADAISLQCWDQEVLLPGGHGLMVRGYRPVINTLAKGLDIRLGHRVVKIV

RHWNRVEVTVSSGKTFVADAAVVAVPLGVLKANTIKFEPRLPEWKEEAIRELSVGVENKI

VLHFSEVFWPNVEFLGVVSSTTYGCSYFLNLHKATGHPVLVYMPAGRLACDIEKMSDESA

AQFAFSQLKKILPNAAEPINYLVSHWGSDENTLGSYTFDGVGKPRDLYEKLRIPVDNLFF

AGEATSVQYTGTVHGAFSTGEMAAEECRMRVLEKFRELDMLEMCHPMAEQTATVSVPLLI

SRL

>BdPAO3-BRADI_5g25730v3-spermine:oxygen_oxidoreductase_(spermidine-forming)_activity

MDPNSFMTGGLLLPTIERRCASPPSVIVIGGGISGIAAARVLSNSSFEVTVLESRDRIGG

RVHTDYSFGCPIDMGASWLHGVSNENSLAPLIGHLGLRLYQTSGDNSVLYDHDLESCSLF

DKNGVQVPRETAAKVGKVFERILEETVKLRDEQEHDMPLQQAISIVLERHPHLKLQGLDD

RVLQWCVCRLEAWFAADADEISLKNWDQEHVLTGGHGLMVDGYYPVVQALARGLDIRLNQ

RVTKVSRQHNRVTVTIEDGTQHCADACIITVPLGVLKANIIKFEPELPLWKSSAIADLGV

GIENKVAMHFDRAFWPNVQVLGMVGPTPKTCGYFLNLHKATGNPVLVYMAAGRFAQEVEK

LSDKEALDIVMSHLKKMIPAAPEPTQYLVSRWGSDPNSLGSYSCDLVGKPADVCERFSAP

VENLYFAGEAASAEHSGAVHGAYSSGLAAAEDCRKRLMLQKGVPDLVQVAACEEVASAAA

EVVAPFQICGT

>BdPAO4-BRADI_5g25740v3-2_splicevariants-spermine_and_thermospermine catabolic process

MDQPPNGFAAGGLFVQHIDGKNASAPSVIVIGGGISGIASARALSNASFKVTLLESRDRL

GGRVHTDYSFGCPIDLGASWLHGVCNENSLAPLIRLLGLRLYRTSGDNSVLYDHDLESYA

LFDKDGRQVPQEIVTKVGETFEQILKETVKVRDEYTNDMPLVQAISIVLDRNPHLKLEGL

QYEVLQWCICRLEAWFATDVDNISLKNWDQEHVLTGGHGLMVNGYDPVIKALARDLDIHL

NHRVTKIIQRYNKVIVCVEDGTSFVADAAIITVPLGVLKANIIKFEPELPDWKLSAISDL

GVGLENKIALRFDTIFWPNVEVIGRVAQTSNSCGYFLNLHKATGHPVLVCMVAGRLAYEM

EKLSDEESVEFVMSQLKRMLPGATEPVQYLVSRWGTDPNSLGSYSCDLVGKPADLYERFC

APVGNLFFAGEAACIDHSGSVHGAYSSGIGAAEDCRRRLSTQLGISDLFQVGKIVMREEM

ADAMVPLQISRL

>BdPAO5-BRADI_2g47960v3-thermospermine oxidase activity-cytoplasm-(IEA)

MVANKPRIVIVGAGIAGLSAAQQLCRAGQGDKFDVVVVEAAPRAGGRVFTSEFAGHRVEM

GATWVQGITGSPVYALAHDAGALTEDAGGHLPYERMDGPFPDDRVLTVAEGGDVVDADKV

AKPVEELYRGMMDAARAGEAGGGGGGVEEYLRRGLRAYQAARTDGSGSKVKEVEEALLAM

HINRERTDTSADALGDLDLAAEGEYRDFPGDHVTIPGGYTRVVEHLVAALPPGTVRLGLR

LRRLDWGETPVRLHFADDGAAALIADHVILTVSLGVLKASLGKDAASAAGAIAFDPPLPQ

FKRDAVSRLGFGVVNKLFVELEAVEPEGGGEEQQLAGAAAPPDFPFLHMAFDGHVAKIPW

WMRGTESICPVHAGSSVALAWFAGREAKHLEFLPDDDVVRGVQATLDSFLPATSSSGATS

RWRVKRIERSRWAGDPLFVGSYSYVAVGSSGGDLDRMAEPLPRGGVPEADRTPPPLRVLF

AGEATHRTHYSTTHAAYLSGVREADRLLQHYP

>HvPAO1-HORVU4Hr1G071450.1-8_splicevariants_oxidoreductase activity

QIGMLLPFAHRCSLYRCSITPLRVTRLACPFAHRRRPNSLPALRLPSVYIKSGWSGEEAI

VCDERLVSTTTRDMKPTTATAALVLALTLAHHASIAAAAGPRVIIVGAGMSGISAGKRLS

EAGITDLVILEATDHVGGRMHKQSFGGINVEVGANWVEGVNGAGRMNPIWPLVNSTLKLK

NFRSDFDGLADNVYKENGGVYERAYVQKRLDRWGEVEEGGEKLSAKLRPSGQDDMSILAM

QRLNDHLPNGPTSPVDMVLDYFKHDYEFAEPPRVTSLQNVVPLATFTDFGDDVYFVADQR

GYEAVVYYLAGQYLKADKSGNIVDPRLQLNKVVTEISHSGGGVTVRTEDAKVYKADYVMV

STSVGVLQSDLIQFKPRLPTWKVLSIYQFDMAVYTKIFVKFPRKFWPQGKGREFFLYASS

RRGYYGVWQEFEAQYPDANVLLVTVTDDESRRIEQQSDNQTKAEIVEVLRSMFPGEDVPD

ATDILVPRWWSDRFYRGTFSNWPIGVNRYEYDQLRAPVGRVYFTGEHTSEHYNGYVHGAY

LSGIDSADILIKCAQKRMCKYHIPGKFD

>HvPAO2-HORVU7Hr1G090410.1-5_splicevariants

MKPSFVTAIAALLLIAAQHASIVAAGKGPRVIIVGAGMSGISAGKRLWDAGVRDLLILEA

TDRVGGRMHKHNFGGLNVEIGANWVEGLNGDKTNPIWPMVNSTLKLRNFYSDFDGVVANV

YKESGGLYDEEFVQKRMDRADEVEELGGKFAAKLDPSGRDDISILAMQRLFNHQPNGPTT

PVDMALDYYKYDYEFAEPPRVTSLQGTEPTATFADFGDDANFVADQRGFETIIYHIAGQY

LRSDKSGNIIDPRVKLNKVVRQISYNDKGVVVTTEDNSAYSADYVMVSTSLGVLQSDLIQ

FKPQLPAWKIMAIYRFDMAVYTKIFLKFPKKFWPTGPGKQFFVYASSRRGYYGMWQSFEK

EYPGANVLLVTVTDVESRRIEQQPDNVTMAEAVGVLRNMFPDRDVPDATDIYVPRWWSNR

FFKGSYSNWPIGVNRYEYDQLRAPVGGRVYFTGEHTSEHYNGYVHGAYLAGIHSADILMN

KALNNVDFKVRPKYDDELKAEAK
