# Supplementary figures and images for: Genome-wide analysis of the polyamine oxidase gene family in wheat (Triticum aestivum L.) reveals involvement in temperature stress response
Source: PLoS One. 2020 Aug 31;15(8):e0236226. doi: 10.1371/journal.pone.0236226 (PMC7458318; doi:10.1371/journal.pone.0236226)

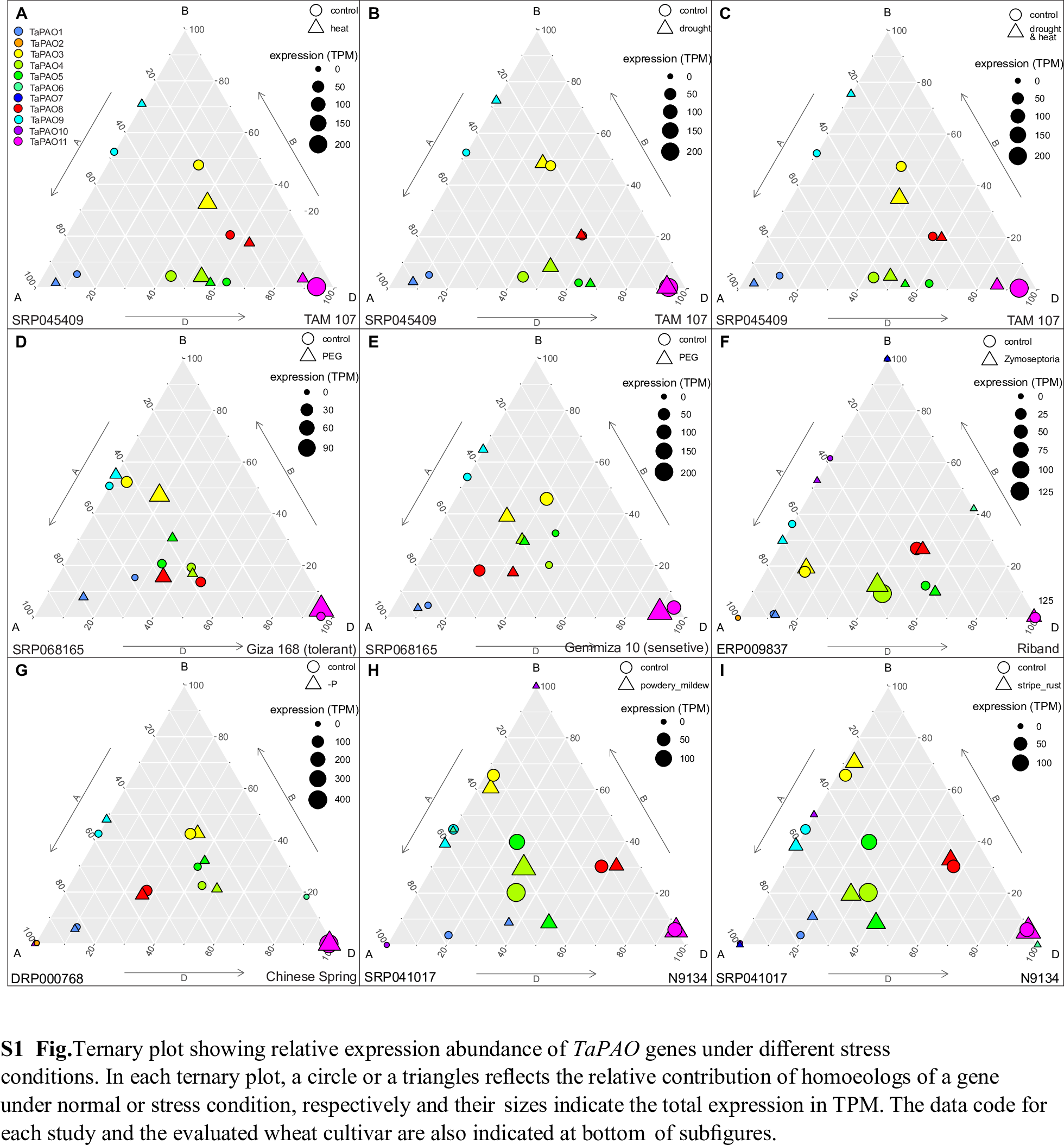

Supplement: S1 Fig — Ternary plot showing relative expression abundance of TaPAO genes under different stress conditions. In each ternary plot, a circle or a triangles reflects the relative contribution of homoeologs of a gene under normal or stress condition, respectively and their sizes indicate the total expression in TPM. The data code for each study and the evaluated wheat cultivar are also indicated at bottom of subfigures. (TIF) [file pone.0236226.s002.tif]

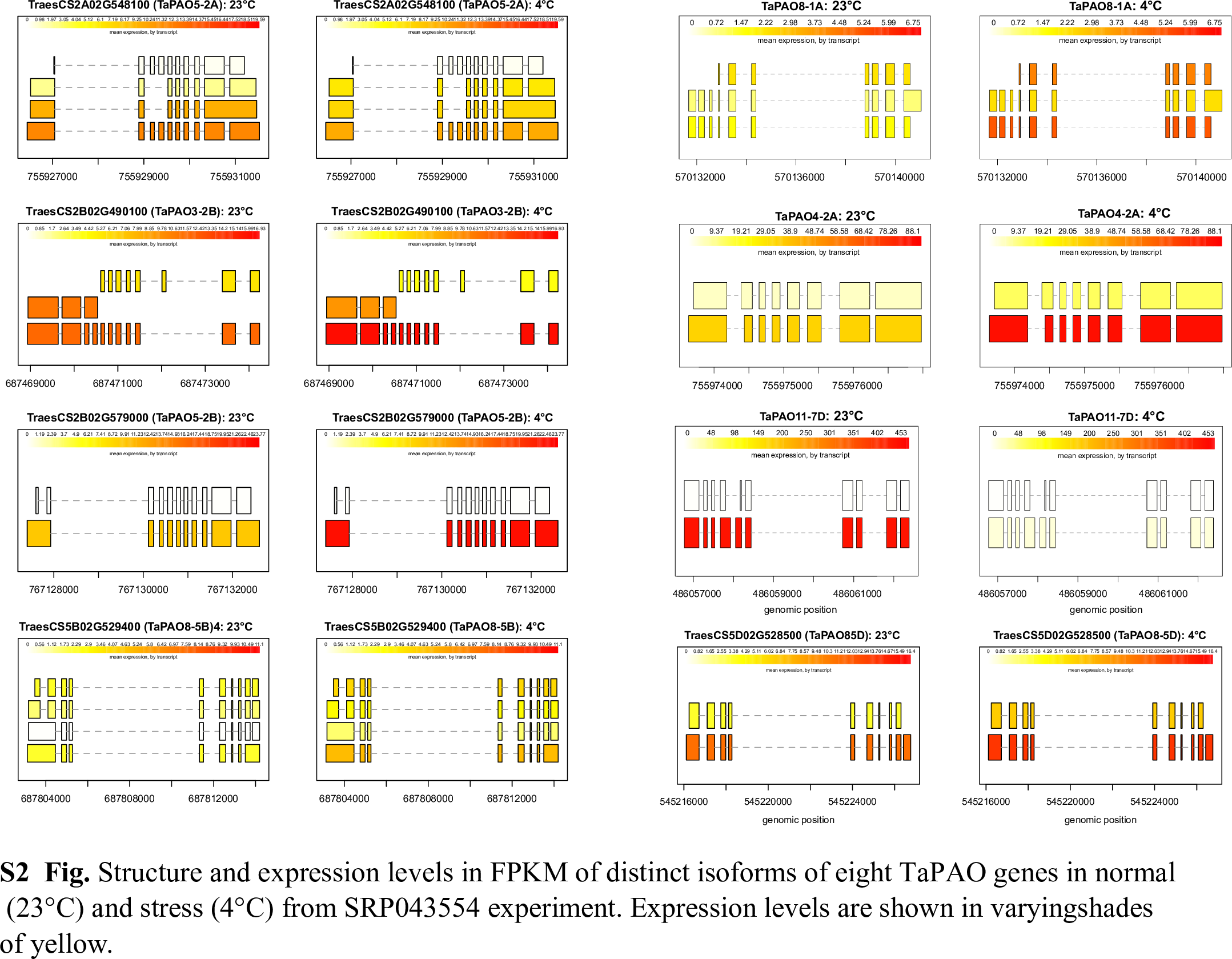

Supplement: S2 Fig — Structure and expression levels in FPKM of distinct isoforms of eight TaPAO genes in normal (23°C) and stress (4°C) from SRP043554 experiment. Expression levels are shown in varying shades of yellow. (TIF) [file pone.0236226.s003.tif]

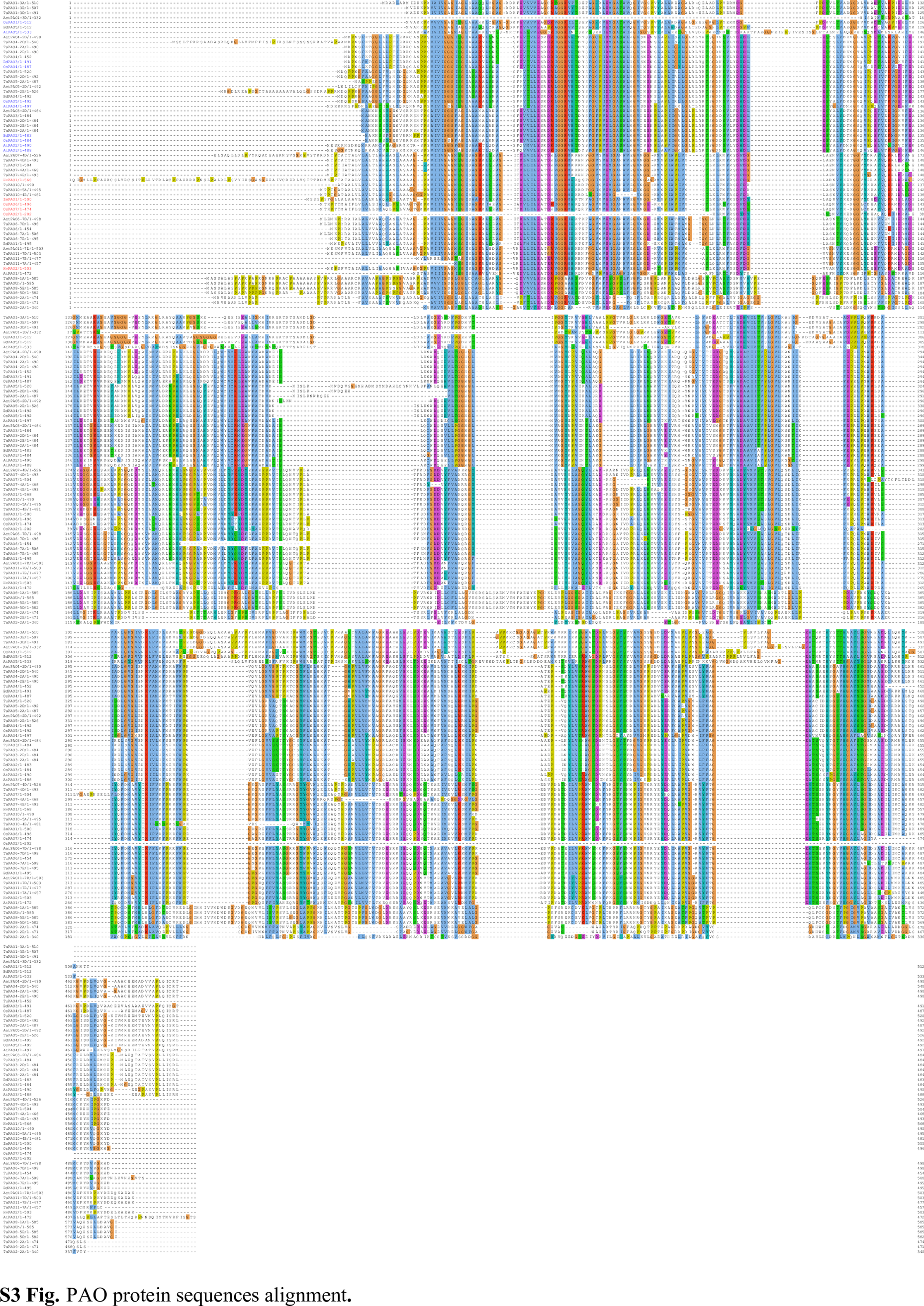

Supplement: S3 Fig — Alignment of the amino acid sequences of PAOs from T. aestivum, T. urartu, Ae. tauschii, A. thaliana, O. sativa, B. distachyon, H. vulgare and Z. mays. The alignment was performed by ClustalW (https://www.genome.jp/tools-bin/clustalw) and exhibited by the Jalview (https://www.jalview.org/). (TIF) [file pone.0236226.s004.tif]
